# Narrative review of models and success factors for scaling up public health interventions

**DOI:** 10.1186/s13012-015-0301-6

**Published:** 2015-08-12

**Authors:** Andrew J. Milat, Adrian Bauman, Sally Redman

**Affiliations:** School of Public Health, University of Sydney, Sydney, Australia; Centre for Epidemiology and Evidence, New South Wales Ministry of Health, 73 Miller St North Sydney, North Sydney, NSW 2060 Australia; Sax Institute, Sydney, Australia

## Abstract

**Background:**

To maximise the impact of public health research, research interventions found to be effective in improving health need to be scaled up and delivered on a population-wide basis. Theoretical frameworks and approaches are useful for describing and understanding how effective interventions are scaled up from small trials into broader policy and practice and can be used as a tool to facilitate effective scale-up. The purpose of this literature review was to synthesise evidence on scaling up public health interventions into population-wide policy and practice, with a focus on the defining and describing frameworks, processes and methods of scaling up public health initiatives.

**Methods:**

The review involved keyword searches of electronic databases including MEDLINE, CINAHL, PsycINFO, EBM Reviews and Google Scholar between August and December 2013. Keywords included ‘scaling up’ and ‘scalability’, while the search terms ‘intervention research’, ‘translational research’, ‘research dissemination’, ‘health promotion’ and ‘public health’ were used to focus the search on public health approaches. Studies included in the review were published in English from January 1990 to December 2013 and described processes, theories or frameworks associated with scaling up public health and health promotion interventions.

**Results:**

There is a growing body of literature describing frameworks for scaling health interventions, with the review identifying eight frameworks, the majority of which have an explicit focus on scaling up health action in low and middle income country contexts. Key success factors for scaling up included the importance of establishing monitoring and evaluation systems, costing and economic modelling of intervention approaches, active engagement of a range of implementers and the target community, tailoring the scaled-up approach to the local context, the use of participatory approaches, the systematic use of evidence, infrastructure to support implementation, strong leadership and champions, political will, well defined scale-up strategy and strong advocacy.

**Conclusions:**

Effective scaling up requires the systematic use of evidence, and it is essential that data from implementation monitoring is linked to decision making throughout the scaling up process. Conceptual frameworks can assist both policy makers and researchers to determine the type of research that is most useful at different stages of scaling up processes.

**Electronic supplementary material:**

The online version of this article (doi:10.1186/s13012-015-0301-6) contains supplementary material, which is available to authorized users.

## Introduction

The transfer of new knowledge from research into policy and practice continues to be sub-optimal [1]. A major reason for slow progress is what some call the “know-do gap”—the gap between what is known in research and what gets implemented [2].

Both the failure of effective public health initiatives to influence public health practice and the lag between evidence generation and implementation represent a considerable impediment to population-wide health improvement, as it denies or delays community access to effective services [3–5]. Even where there is evidence of the efficacy or effectiveness of public health interventions, there has been much less attention paid to the mechanisms for delivering them to scale [6].

Scaling up is the process by which health interventions shown to be efficacious on a small scale and or under controlled conditions are expanded under real world conditions into broader policy or practice [7]. The concept of scaling up is different from routine adoption as it involves an explicit intent to expand the reach of an intervention to new settings or target groups and is accompanied by systematic strategy to achieve this objective [8].

The issue of how best to scale up health interventions has been receiving recent attention, particularly in the global health literature [7, 9] but there are few studies that offer frameworks and methods for the effective scale up of public health interventions [10].

Theoretical frameworks and approaches are useful for describing and understanding how effective interventions are scaled up from small trials into broader policy and practice and can be important support tools for policy makers and practitioners in their efforts to scale up public health interventions. Such evidence-to-practice frameworks are gaining greater prominence in public health and health care [7].

The purpose of this literature review was to synthesise evidence on scaling up public health interventions into population-wide policy and practice, with a focus on defining and describing the processes and frameworks that support the scale up of public health initiatives. The review also aimed to identify key success factors and barriers to effective scale up of public health interventions.

## Methods

### Literature review search strategy

The review included publications with a focus on concepts, theories and models for scaling up of public health interventions. For the purposes of this review, an *intervention* was defined as a set of actions with a coherent objective to bring about change or produce identifiable outcomes [10]. These actions may include policy, regulatory initiatives, single strategy projects or multi-component programmes. *Public health interventions* are intended to promote or protect health or prevent ill health in communities or populations, and they are distinguished from clinical interventions, which are intended to prevent or treat illness in individuals [10]. Table 1 details the relevant study descriptions, search terms and databases covered in the review.

**Table 1:** Study designs, review search terms and databases used in the literature review of scaling up public health action

Study descriptions	Review search terms	Review databases
Theoretical and opinion pieces	Scaling up OR	MEDLINE (general medicine)
	Scalability OR	CINAHL (nursing and allied health)
Case studies	Intervention research OR	
	Translational research OR	EBM reviews—Cochrane database of systematic reviews 2005 to December 2013
Descriptive studies	Research dissemination OR	
Intervention studies	Health promotion AND	
Frameworks	Public health AND	
		PsycINFO (psychology and related behavioural and social sciences)
Systematic reviews		
		Google Scholar

The review was conducted in two phases between August and December 2013. In phase 1, abstracts were retrieved and assessed against the review criteria. For abstracts that met the review criteria in phase 1, full papers were retrieved and were assessed against the review criteria. Studies included in the review met the following criteria:

- Published in English from January 1990 to December 2013
- Described processes, theories or models/frameworks associated with scaling up public health and health promotion interventions
- Were theoretical and opinion pieces, case studies, descriptive studies or intervention studies

For the purpose of this review, ‘concepts’ were defined as scientific methods that provide information that informs the scale up of interventions such as epidemiological forecasting, economic methods etc. ‘Theories’ were defined as ideas that explain scale up and offer principles upon which scale up can be based. Finally, ‘frameworks/models’ propose a structure around which scale-up can be organised. Frameworks/models are used to inform decisions and judgments about scaling up and often apply multiple concepts, processes and theories.

**Fig. 1:**
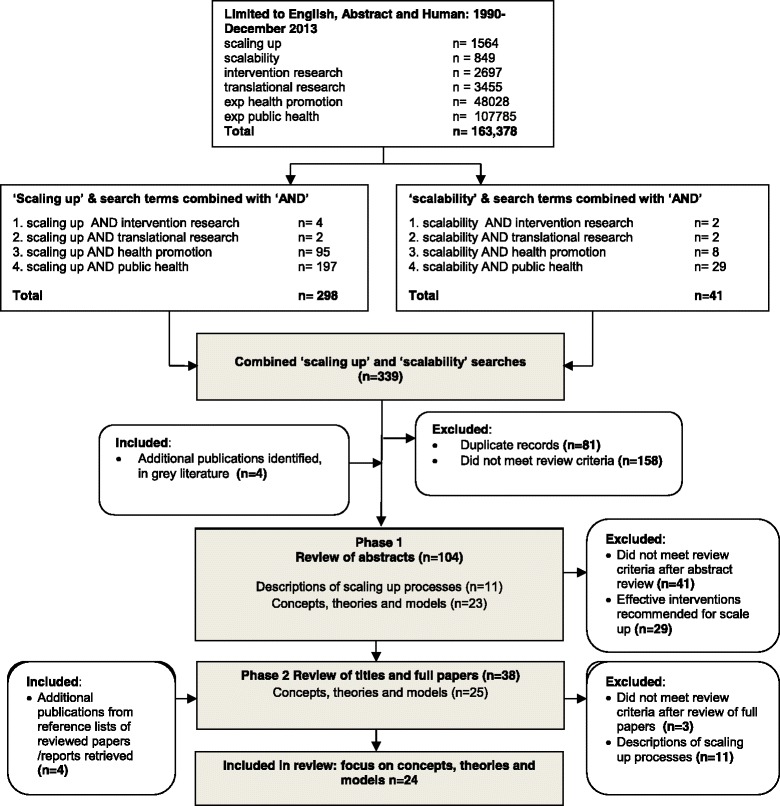
Literature search process and numbers of papers identified, excluded and included in the review of increasing the scale of public health interventions

Studies focusing on the scaling up of health services and public health or health promotion programmes were included in the review, while studies with a sole focus on health services were excluded. In this review, ‘health services’ referred to the provision of clinical care in hospital and community settings. The reference lists of the full papers were also checked, and relevant papers were included in the final review. The search process is summarised in Fig. 1.

The full papers were also reviewed and thematically analysed [11] to determine key success factors and barriers to scale-up. The papers were reviewed and a code frame developed by the lead author in discussion with co-authors. The lead author then recorded the frequency of mentions of success factors and barriers.

## Results

### Findings of the literature review

#### Search results

An initial review of abstracts in electronic databases against the inclusion criteria yielded 100 abstracts, and searches of the grey literature further identified four documents. Of the 104 papers and documents reviewed against the inclusion criteria, 29 papers recommended effective interventions for scaling up, but did not examine concepts, theories and models in any great detail (so were excluded), 11 papers provided accounts of scaling up processes of public health interventions and 25 papers described concepts, theories and models relevant to scaling up public health interventions. The majority of papers excluded from phase 1 of the review (*n* = 41) focused on health service interventions, particularly expanding anti-retroviral treatment for HIV in low income countries.

In phase 2 of the review, a total of 38 full papers and reports were retrieved and reviewed against the inclusion criteria (*n* = 38), with 11 papers providing accounts of scaling up processes of public health interventions. Twenty-five papers and reports described concepts, theories and models relevant to scaling up public health interventions, of which 24 were included in the final review.

Additional file 1: Table S1 summarises characteristics of papers and reports focusing on concepts, theories and models relevant to scaling up public health interventions including reference details, study type, concepts, theories used or proposed, key concepts, elements of the model or framework, context and success factors and important findings. A number of studies were both case studies and proposed theoretical frameworks for scaling up public health interventions and were described as such in the ‘study type’ column of Additional file 1: Table S1.

#### Study characteristics

The literature on processes, theories or frameworks associated with scaling up public health and health promotion interventions is growing rapidly, with 21 of the 24 (88 %) publications in this review published since 2006, highlighting that this is a new field of public health enquiry. Research in this field continues to be characterised by great variation in definitions, processes and models. Studies in this review could be grouped under three broad themes: economic and mathematical models and methods [12–15]; scaling up theories and principles [16–22]; and scaling up frameworks (both generic and issue specific) [7, 9, 23–28].

#### Defining scaling up and scalability

Mangham and Hanson [18] suggest that scaling up is used primarily to describe the ambition or process of expanding the coverage of health interventions, but can also refer to increasing the financial, human and capital resources required to expand coverage. WHO and ExpandNet define scaling up as ‘…deliberate efforts to increase the impact of successfully tested health innovations so as to benefit more people and to foster policy and program development on a lasting basis’ [see p. 2 in 7]. The term ‘scaling up’ has been applied in the literature in several distinct ways including to describe the following: the dissemination of a new technique, a prototype product, or process innovation [16, 17, 21, 27, 29], epidemiological and economic forecasting; [13–15, 30, 31] ‘growing’ an organisational or system capacity to implement to a new level [29, 32–34]; and translating a small-scale initiative into a government policy [35–37].

The papers included in this review offered only one definition of the term ‘scalability’, which was developed by the authors of this manuscript in 2011 using an expert Delphi process with Australian policy makers and researchers as follows: [25, 26, 35] ‘…the ability of a health intervention shown to be efficacious on a small scale and or under controlled conditions to be expanded under real world conditions to reach a greater proportion of the eligible population, while retaining effectiveness’ [25].

#### Frameworks for scaling up public health interventions

The review identified eight frameworks [7, 9, 23–28] (see Table 2), the majority of which had an explicit focus on scaling up health action in low- and middle-income countries, indicating a gap in scaling up methods in high-income country contexts. Of these, three frameworks focused on scaling up specific health interventions [23, 27, 28] (i.e. insecticide-treated nets for malaria, promoting breastfeeding and maternal nutrition programmes), while five propose generic frameworks that can be applied to efforts to scale up a range of public health endeavours [7, 9, 24–26]. Generic frameworks will be examined more closely as they have the greatest potential to guide public health action across many areas.

The oldest of these generic frameworks, the scaling up management (SUM) framework, was developed by Kohl and colleagues in 2003, and was updated in 2012 [24, 38]. It proposes three key steps with a series of tasks under each step. The first step involves developing a scaling up plan and creating a vision of what scaling up will look like if successfully implemented. Step 2 involves establishing the preconditions for scaling up, with key tasks including building the legitimacy of the intervention and the proposed approach, constituency building and realigning and mobilising resources. In the final step, the scaling up process is implemented based on the identification of factors that can promote extension and sustainability. Key tasks involve modifying organisational structures, coordinating action and performance monitoring.

The WHO and ExpandNet (2010) [7] model developed by Simmons, Ghiron, Fajans and Newton based on earlier work by Simmons and Shiffman (2007) [39] offers a slightly different way of systematically thinking about scaling up and was developed in light of the literature and expert opinion. The conceptual model accompanying the framework consists of five elements, with the scaling-up strategy as the centrepiece and five strategic choice areas (dissemination and advocacy; organisational process; cost and resource mobilisation; monitoring and evaluation). The framework is guided by four key principles which are systems thinking; a focus on sustainability; the need to determine scalability; and respect for gender, equity and human rights principles.

The framework proposes nine steps for developing a scaling-up strategy that involve the following: i) planning actions to increase the scalability of the innovation; ii) increasing the capacity of the user organisation to implement scaling up; iii) assessing the environment and planning actions to increase the potential for scaling-up success; iv) increasing the capacity of the resource team to support scaling up; v) making strategic choices to support vertical scaling up (policy, political, regulatory, resourcing or other health systems changes needed to institutionalise the innovation); vi) making strategic choices to support horizontal scaling up (replicating innovations in different geographic sites or extending them to serve larger or different population groups); vii) determining the role of diversification; viii) planning actions to address spontaneous scaling up; and ix) finalising the scaling-up strategy and identifying next steps.

**Table 2:** Synthesis of public health scaling up models and frameworks

Model/framework	Key components	Focus area	Process of development	Context	Bibliographic references
Scalability considerations	Scalability with a focus on the following:	Health promotion: generic	Literature review and expert Delphi process	High-income country	[25]
	➢ Effectiveness				
	➢ Reach and adoption				
	➢ Human, technical and organisational Resources				
	➢ Costs				
	➢ Intervention delivery				
	➢ Contextual factors				
	➢ Appropriate evaluation approaches				
Scaling up population health interventions: guide, New South Wales Ministry of Health	A 4-step process for scaling up interventions:	Public health: generic	Literature review and expert Delphi process	High-income country	[26]
	➢ Step 1. Scalability assessment: to assess the suitability of the intervention/s for scaling up				
	➢ Step 2. Develop a scaling up plan: create a vision of what scaling up will look like and a compelling case for action				
	➢ Step 3. Prepare for scaling up: securing resources and building a foundation of legitimacy and support for the scaling up plan				
	➢ Step 4. Scale up: the main tasks that should be addressed during scale up				
9 steps to scaling up, WHO ExpandNet	ExpandNet framework involves 9 steps:	Health services and public health: generic	Literature review and interviews	Global health	[7, 39]
	➢ Planning actions to increase the scalability of the innovation				
	➢ Increasing the capacity of the user organisation to implement				
	➢ Assessing the environment and planning actions to increase the potential for success				
	➢ Increasing the capacity of the resource team to support scaling up				
	➢ Making strategic choices to support vertical scaling up (institutionalisation)				
	➢ Making strategic choices to support horizontal scaling up (expansion/replication)				
	➢ Determining the role of diversification				
	➢ Planning actions to address spontaneous scaling up				
	➢ Finalising the scaling-up strategy and identifying next steps				
Scaling up management (SUM) framework	Includes 3 key steps:	Health services and public health: generic	Literature and interviews	Global health	[24]
	➢ Step 1: developing a scaling up plan				
	➢ Step 2: establishing the preconditions for scaling up				
	➢ Step 3: implementing the scaling up process based on the identification of factors that can promote extension and sustainability				
Scale up of exclusive breastfeeding	Involves the following steps:	Health services and public health: breastfeeding	Systematic review	Global health	[23]
	➢ Assess situation, create a policy environment conducive to action				
	➢ Define roles, relationships and responsibilities of all partners				
	➢ Establish agreements				
	➢ Review technical support				
	➢ Define programme strategy				
	➢ Mobilise resources				
	➢ Provide training and technical assistance				
	➢ Develop and use monitoring and evaluation systems				
	➢ Monitor coverage and quality				
	➢ Measure impact and cost				
	➢ Provide for testing novel approaches and continuing innovation				
Scaling up global health interventions: framework for success	Describes 6 components of the scaling up process:	Health services and public health	Literature review and interviews with experts	Global health	[9]
	➢ Attributes of the specific tool or service being scaled up				
	➢ Attributes of the implementers				
	➢ Chosen delivery strategy				
	➢ Attributes of the ‘adopting’ community				
	➢ Socio-political context and research context				
Breastfeeding gear model	Eight interrelated elements:	Health services and public health	Literature review, interviews and focus groups	Global health	[27]
	➢ Advocacy				
	➢ Political will				
	➢ Legislation and policies				
	➢ Funding and resources				
	➢ Training and delivery				
	➢ Promotion				
	➢ Research and evaluation				
	➢ Coordination and goals monitoring				
	The framework suggests successful multiple feedback loops and several potential paths are required to achieve intended innovation				

Yamey (2011) [9] offers a framework and key success factors for scaling up global health initiatives based on a literature review and interviews with ‘thought leaders’. Yamey’s framework divides the scaling up process into six categories: attributes of the specific tool or service being scaled up; attributes of the implementers; the chosen delivery strategy; attributes of the ‘adopting’ community; the socio-political context; and the research context.

Each of Yamey’s categories will now be examined in greater detail, starting with the attributes of the specific tool or service being scaled up. Keeping the intervention simple is considered a predictor of success [40, 41], and technical experts who have managed large-scale implementation also argue that getting the implementation policies and procedures scientifically robust and evidence informed before going to scale is crucial for success [42].

Addressing the attributes of the implementers, the framework suggests that strong leadership and governance play an important role in successful scale up as is getting buy-in from local implementers and other key stakeholders. The framework also recommends using both state and non-state actors as implementers.

The chosen delivery strategy is also of great importance, with the framework recommending the application of diffusion of innovation theory by focusing on the five factors identified by Rogers (1995) [43] as being positively associated with the faster diffusion of an innovation. The framework also describes cascade and phased approaches to scaling up depending on the context within which an intervention is delivered. Cascade approaches use a ‘train the trainer’ approach that can result in rapid expansion of interventions. Going to scale in a phased manner begins with a pilot programme, followed by stepwise expansion and learning lessons along the way to help refine further expansion. Tailoring scale-up to the local situation and decentralising delivery by adopting an integrated approach to scale-up is also considered important.

Thinking about the attributes of the adopting community can be facilitated through the active participation of the community in planning, implementing, and monitoring interventions and is cited as a crucial factor in successful scale-up. Being cognisant of the socio-political context is a vital consideration in the framework, particularly building political good will and alignment with national policies. Finally, the framework requires due consideration of the research context. This can be done by incorporating research into implementation using ‘learning and doing’ approaches that involve the systematic application of evidence to guide the process and incorporate new learning.

Milat and colleagues developed a model of ‘scalability considerations’ using a literature review and expert Delphi process in 2012 [25], which was further developed in 2013 into the ‘increasing the scale of population health interventions guide’ [26] for the NSW Ministry of Health in Australia. The framework proposes a four-step process for scaling up interventions. It differs from other models discussed as it is specifically designed for scale-up of public health interventions in high income countries and is unique in that step 1 involves a ‘scalability assessment’ that determines the suitability of the intervention/s by assessing effectiveness, potential reach and adoption, alignment with the strategic context and intervention acceptability and feasibility. The outcome of this assessment will determine whether the remaining steps in the guide should be followed.

Step 2 describes how to develop a scaling up plan which should create a vision of what scaling up will look like and a compelling case for action. This step involves documenting a rationale for scaling up, describing the intervention, completing a situational and stakeholder analysis, determining who could be involved in scale up and what their role will be, selecting an approach to scaling up, considering options for evaluation and monitoring and estimating resources required for scale up and writing up the plan.

Step 3 describes how to prepare for scaling up by securing resources and building a foundation of legitimacy and support for the scaling up plan process. This step involves consultation with stakeholders, legitimising change, building a broad constituency and realigning and mobilising resources. Finally, step 4 describes the main tasks that should be addressed during scaling up including modifying and strengthening organisations, coordinating action and governance, monitoring performance, quality and efficiency and ensuring sustainability.

Though many of these frameworks propose linear processes for scaling health interventions, it is acknowledged by their authors that the reality of ‘real world’ scale-up is that one or more steps in scale up are often missed. For example, using the Milat et al. 2014 model as an example, initiatives often go from a scalability assessment (step 1) to full implementation (step 4), without establishing important preconditions for success such as building a broad constituency and realigning and mobilising resources [8, 19, 26].

A common characteristic of scaling up models identified in this review is that they link many existing concepts in the literature and interpret them together and in relation to one another to illuminate factors that inform large-scale implementation of public health interventions. Common characteristics of these models include a focus on understanding the attributes of the intervention being scaled up (effectiveness, potential reach, acceptability etc.), identifying and supporting implementers, the selection of an appropriate delivery strategy, understanding and accommodating the characteristics of the adopting community, taking into account the broader socio-political context, and the use of research, evaluation and monitoring data to inform the scale-up process. Importantly, these frameworks enable a clearer discourse and common understanding of key concepts and methods associated with the scale-up of public health interventions.

#### Success factors and barriers to effectively scaling up public health interventions

Key success factors for scaling up health interventions gleaned from this review, particularly from case studies and papers that interviewed implementation experts in order of frequency of mention in the literature are the following: establishing monitoring and evaluation systems [5, 7, 11, 13, 17, 20, 25–27, 29–31, 44]; costing and economic and other modelling of intervention approaches [10, 12, 13, 16–18, 20, 23, 24, 28, 32, 44]; active engagement of a range of implementers and the target community [7, 9, 13, 17, 20, 25–27, 29, 31, 44]; tailoring the scale-up approach to the local context and use of participatory approaches [5, 7, 13, 17, 26, 29, 41, 44]; systematic use of evidence [7, 9, 13, 17, 26, 31, 44]; infrastructure to support implementation such as training, delivery systems, technical resources [13, 17, 20, 27, 31, 32, 44], strong leadership and champions [7, 9, 13, 17, 25, 44]; political will [7, 9, 17, 25, 32]; well defined scale up strategy [9, 13, 24, 27, 44]; and strong advocacy [7, 9, 12, 22, 27] (See Table 3).

There is merit in more closely examining some of these success factors starting with the importance of the use of evidence. It was widely noted in papers that effective scale-up requires the systematic use of different types of evidence. For example, Simmons and Shiffman [39] argue that successful scale-up ‘…requires the systematic use of evidence to guide the process and incorporate new learning.’ It was also noted that quality control and performance monitoring systems should replace stand-alone evaluation as interventions increase further in scale and are disseminated widely into policy and practice.

**Table 3:** Synthesis of success factors and barriers to scaling up public health interventions in rank order of mentions

Success factors	Bibliographic references
Establishing monitoring and evaluation systems	[7, 9, 16, 17, 19–22, 24–27, 29]
Costing and economic modelling of intervention approaches	[12, 13, 15, 18, 22, 24–26, 28, 30, 31, 44];
Active engagement of a range of implementers and the target community	[9, 19–27, 29];
Tailoring scale-up approach to local context and use of participatory approaches	[7, 9, 20, 24–26, 29, 45];
Systematic use of evidence	[9, 23–27, 29]
Infrastructure to support implementation such as training, delivery systems, technical resources	[21, 22, 24–28]
Strong leadership and champions	[9, 19, 23–26]
Political will	[9, 19, 23, 25, 28]
Well-defined scale-up strategy	[9, 13, 19, 21, 23–26]
Strong advocacy	[9, 23, 28, 29]
Flexible responses to human resource constraints	[18, 25, 26, 46]
Formative research to ensure appropriate intervention design	[23, 25–27]
Equity of intervention delivery and monitoring intended and unintended consequences across socio-demographic profiles	[17, 25, 26, 28]
Effective communication strategy	[18, 21, 23, 27]
Effective governance and coordination	[9, 26, 29]
Clear role definition and delineation	[17, 23, 26]
Keeping the intervention model simple	[9, 24, 26]
Financing models	[20, 21, 28]
Programmes are visible, publicised and effectively packaged	[19, 25]
Developing strategies for integration into existing services	[19, 21, 26]
Barriers	
Not adapting intervention approaches to the local context	[18–20, 24]
Intervention costs and other economic factors	[12, 22, 25, 32]
Lack of human resources	[13, 18, 19]
Resistance to the introduction of new practices due to capacity constraints	[18, 19, 26]
Insufficient investment in implementation infrastructure including training, monitoring and evaluation systems	[17, 18, 45]
Staff recruitment and staff turnover	[18, 19, 46]
Lack of political will	[9, 32]
Traditional research funding processes are not flexible enough to support evaluation of scale up	[19]
Leadership changes amongst implementation agencies	[19]
Poor engagement with stakeholders and thought leaders	[52]
Poor role delineation	[32]
Maintaining quality and consistency of health interventions at scale [18]	[18]

Information on programme costs and other economic considerations were considered fundamental to making effective decisions about the appropriateness and feasibility of population-level programme implementation. Failure to address economic outcomes was often noted as a barrier to scale-up and the presence of this data was conversely considered an important facilitator of effective decision making.

Consideration of the context within which interventions are delivered was widely identified as an important success factor. Tailoring the scale-up approach to the settings within which they operate such as community characteristics, financial and human resources and local socio-political landscape was thought to be facilitated by the use of participatory approaches that include active engagement of a range of implementers and the target community.

Barriers to successful scale-up of public health interventions identified in the review were often the converse of the success factors and ranged from the following: not adapting intervention approaches to the local context [18–20, 24], intervention costs and other economic factors [12, 22, 25, 32], lack of human resources [13, 18, 19], resistance to the introduction of new practices due to capacity constraints [18, 19, 26], insufficient investment in implementation infrastructure including training, monitoring and evaluation systems [17, 18, 45], staff recruitment and staff turnover [18, 19, 46] and lack of political will [9, 32].

There were a number of challenges identified in the literature to moving interventions from a ‘research’ phase to a widespread adoption or scaling up phase in high-income countries. Norton and Mittman’s [19] examination of barriers and enablers to scaling up ten promising health promotion and disease prevention interventions in the USA found that many of the organisations implementing the programmes during initial research phases viewed the programmes as experimental and time-limited, and were reluctant to have interventions become fully integrated into the organisation’s routine service delivery after the study. They also found that a number of research teams were subsequently unable to implement the programme according to original experimental protocol in real world settings using community-based organisations (e.g. senior centres), and had to adapt the interventions to fit typical organisations with limited resources.

## Discussion

While the scale of an intervention may seem an obvious concept, the findings of this review confirm that the terms scaling up and scalability have been applied in different ways and contexts with little consistency. Though scalability is a less frequently used term in the context of public health [25, 35], it appears to have a similar intent to that of scaling up. Clarifying the meaning and relationships between new and emerging terminology is an important endeavour as it facilitates precise communication amongst those working and researching an interdisciplinary field like public health [47].

There is a growing body of literature describing frameworks for scaling health interventions, with the review identifying seven frameworks [7, 9, 23, 24, 26–28], the majority of which have an explicit focus on scaling up health action in low- and middle-income country contexts. While the majority of these frameworks were specifically developed for scaling up in low- and middle-income countries, they are generally applicable to scaling up public health action in high-income contexts as they have a similar focus on improving health status through action directed toward the health of an entire population, or sub-population, rather than individuals. However, population action in high-income countries is characterised by fewer resource and capacity constraints than in global health contexts. In addition, policy makers in high-income countries can bring greater technical and system capacity to bear on public health problems and as such models for scaling up public health action in these contexts should keep this in mind.

The review identified a number of key success factors in efforts to scale up health interventions including strong leadership and governance [9, 28, 29, 32, 37], active engagement of a range of implementers and of the target community [9, 19, 27, 29] and tailoring the scale-up approach to the local context [7, 9, 45].

Costs and economic modelling of scaling up interventions were widely considered fundamental to strategic decisions about public health programme implementation at various stages of scale up [12, 13, 22, 25, 30]. Costing of an intervention identifies whether the various arms of a programme are receiving money as was intended in the original plan and underpins any subsequent economic evaluation [48]. Despite its value, this information is generally absent from research reports and in particular published intervention studies [25, 49]. Given the importance of economic data to informing scaling up processes [25], the field should be encouraged to collect and publish data on intervention costs and where feasible cost effectiveness of interventions.

The notion of keeping the intervention simple as a success factor merits further consideration in light of the complexity of many population health interventions. Complex population health interventions can be multi-level and multi-component in nature and by virtue of this are not simple. However, the literature suggests that though the overall strategy may be complex, individual intervention components that are easy to understand and adopt by key stakeholders and target audiences are more readily scaled up.

Finally, the importance of research, evaluation and monitoring systems to effective scale-up was widely noted as an important success factor [7, 9, 17, 19, 22, 24, 27, 29]. The systematic use of evidence and data from implementation monitoring that is linked to decision making throughout the scaling up process can be of great assistance in scaling processes. It is important that these evaluative and monitoring frameworks are built into intervention delivery from the outset and produce reliable and timely information to inform scaling up decision making [7, 9, 24, 25].

It is also worth noting that three of the generic scaling up frameworks (scaling up management framework and ExpandNet; increasing the scale of population health intervention guide) were identified in the grey literature and were not published in the peer reviewed literature. This highlights the importance of publishing future frameworks in the peer reviewed literature to increase their dissemination to the field.

Giving close consideration barriers and enablers to scaling up interventions in developed country contexts, Norton and Mittman’s [19] examination of the scale up promising health promotion and disease prevention interventions in the USA suggests that interventions should be tested in more typical resource-constrained community-based settings before broader roll out. This view is supported by Glasgow and Emmons [50] and Rychetnik and colleagues [51] who argue that more funding and reporting of practical trials that address external validity and contextual issues will significantly enhance translation into practice.

A limitation of this review was that it did not examine efforts to expand the reach and impact of public health policies and programmes, where studies did not use the terms scaling up and scalability to describe such efforts. This was done to impose some degree of specificity of concepts that might otherwise prove too diffuse for identification in the literature and subsequent analysis. However, there is merit in future reviews of the scaling up literature including search terms such as ‘spread’ and ‘diffusion of innovation’ in order to capture the greatest number of relevant studies. Key success factors and barriers for scaling up health interventions gleaned from this review are listed in order of the frequency of mentions in reviewed papers. These factors were identified using thematic analysis, a method for identifying, analysing and reporting patterns (themes) within data. As the reviewed papers offered empirical observations rather than evidence of causality, caution should be exercised when interpreting the success factors and barriers*.*

Another methodological limitation is that we did not estimate the level of publication bias and selective publication in this emerging field. Finally, our analysis included studies published up to December 2013 and we may not have captured emerging approaches to scaling up health interventions.

## Conclusion

Theoretical frameworks and approaches are useful for describing and understanding how effective interventions are scaled up from small trials into broader policy and practice. There is a growing body of literature describing frameworks for scaling health interventions, most of which have an explicit focus on scaling up health action in low- and middle-income country contexts. Effective scaling up processes requires the systematic use of evidence, and it is essential that data from implementation monitoring is linked to decision making throughout the scaling up process. Conceptual frameworks reviewed can assist both policy makers and researchers in determining the types of research that are most useful at various stages of a scaling up process.

## Additional file

Additional file 1: Table S1. Summary of characteristics of studies describing concepts, theories and models of scaling up public health action.

## References

[1] Productivity Commission (2010). Strengthening evidence-based policy in the Australian Federation, volume 2: background paper.

[2] World Health Organisation. Bridging the “know–do” gap meeting on knowledge translation in global health. http://www.who.int/kms/WHO_EIP_KMS_2006_2.pdf Accessed July 2015.

[3] Sanson-Fisher RW, Campbell EM, Htun AT, Bailey LJ, Miller CJ (2008). We are what we do: research outputs of public health. Am J Prev Med.

[4] McKeon R (2013). Strategic review of health and medical research in Australia—better health through research.

[5] Milat AJ, Bauman A, Redman S, Curac N. Public health research outputs from efficacy to dissemination: a bibliometric analysis. BMC Public Health. 2011, 11:doi:10.1186/1471-2458-1111-1934. 10.1186/1471-2458-11-934 PMC3297537 22168312

[6] Catford J (2009). Advancing the ‘science of delivery’ of health promotion: not just the ‘science of discovery’. Health Promot Int.

[7] World Health Organization (2010). ExpandNet: nine steps for developing a scaling-up strategy.

[8] Milat AJ, King L, Wolfenden L, Rissel C, Newson R, Bauman AE, et al. Increasing the scale and adoption of population health interventions: experiences and perspectives of policy makers, practitioners and researchers. Health Res Policy Syst. 2014, 12(18). 10.1186/1478-4505-12-18 PMC3996855 24735455

[9] Yamey G (2011). Scaling up global health interventions: a proposed framework for success. PLoS Med.

[10] Rychetnik L, Frommer M, Hawe P, Shiell A (2002). Criteria for evaluating evidence on public health interventions. J Epidemiol Community Health.

[11] Boyatzis RE (1998). Transforming qualitative information: thematic analysis and code development.

[12] Kumaranayake L (2008). The economics of scaling up: cost estimation for HIV/AIDS interventions. AIDS.

[13] Maher D, Dye C, Floyd K, Pantoja A, Lonnroth K, Reid A (2007). Planning to improve global health: the next decade of tuberculosis control. Bull World Health Organ.

[14] Winfrey W, McKinnon R, Stover J (2011). Methods used in the lives saved tool (LiST). BMC Public Health.

[15] Zhang L, Gray RT, Wilson DP (2012). Modelling the epidemiological impact of scaling up HIV testing and antiretroviral treatment in China. Sex Health.

[16] Edouard E, Edouard L (2012). Application of information and communication technology for scaling up youth sexual and reproductive health. Afr J Reprod Health.

[17] Larson CP, Koehlmoos TP, DA S (2012). Scaling up zinc treatment of childhood diarrhoea in Bangladesh: theoretical and practical considerations guiding the SUZY project. Health Policy Plan.

[18] Mangham LJ, Hanson K (2010). Scaling up in international health: what are the key issues?. Health Policy Plan.

[19] Norton W, Mittman B (2010). Scaling-up health promotion/disease prevention programs in community settings: barriers, facilitators, and initial recommendations.

[20] Subramanian S, Naimoli J, Matsubayashi T, Peters DH (2011). Do we have the right models for scaling up health services to achieve the millennium development goals?. BMC Health Serv Res.

[21] Underhill K, Operario D, Mimiaga MJ, Skeer MR, Mayer KH (2010). Implementation science of pre-exposure prophylaxis: preparing for public use. Curr HIV/AIDS Rep.

[22] Willey BA, Paintain LS, Mangham L, Car J, Schellenberg JA (2012). Strategies for delivering insecticide-treated nets at scale for malaria control: a systematic review. Bull World Health Organ.

[23] Bhandari N, Kabir AKM, Salam MA (2008). Mainstreaming nutrition into maternal and child health programmes: scaling up of exclusive breastfeeding. Matern Child Nutr.

[24] Kohl R, Cooley L (2003). Scaling up—a conceptual and operational framework.

[25] Milat AJ, King L, Bauman A, Redman S. The concept of scalability: increasing the scale and potential adoption of health promotion interventions into policy and practice. Health Promot Int. 2012:doi:10.1093/heapro/dar1097. 10.1093/heapro/dar097 22241853

[26] Milat AJ, Newson R, King L (2014). Increasing the scale of population health interventions: a guide.

[27] Pérez-Escamilla R, Curry L, Minhas D, Taylor L, Bradley E (2012). Scaling up of breastfeeding promotion programs in low-and middle-income countries: the “breastfeeding gear” model. Adv Nutr.

[28] Victora CG, Barros FC, Assunção MC, Restrepo-Méndez MC, Matijasevich A, Martorell R (2012). Scaling up maternal nutrition programs to improve birth outcomes: a review of implementation issues. Food Nutr Bull.

[29] Pearson BL, Ljungqvist B (2011). REACH: an effective catalyst for scaling up priority nutrition interventions at the country level. Food Nutr Bull.

[30] Johns B, Baltussen R (2004). Accounting for the cost of scaling up health interventions. Health Econ.

[31] Morel CM, Lauer JA, Evans DB (2005). Cost effectiveness analysis of strategies to combat malaria in developing countries. BMJ.

[32] Rani M, Nusrat S, Hawken L (2012). A qualitative study of governance of evolving response to non-communicable diseases in low-and middle-income countries: current status, risks and options. BMC Public Health.

[33] Merson MH, Curran JW, Griffith CH, Ragunanthan B (2012). The president’s emergency plan for AIDS relief: from successes of the emergency response to challenges of sustainable action. Health Aff.

[34] de Silva-Sanigorski AM, Bolton K, Haby M, Kremer P, Gibbs L, Waters E, et al Scaling up community-based obesity prevention in Australia: background and evaluation design of the health promoting communities: being active eating well initiative. BMC Public Health. 2010, 10(65). 10.1186/1471-2458-10-65 PMC2836295 20152018

[35] Marrero D (2009). The prevention of type 2 diabetes: an overview. J Diabetes Sci Technol.

[36] Aldinger C, Zhang XW, Liu LQ, Guo JX, Hai YS, Jones J (2008). Strategies for implementing health-promoting schools in a province in China. Promot Educ.

[37] Nankunda J, Tylleskär T, Ndeezi G, Semiyaga N, Tumwine JK (2010). Establishing individual peer counselling for exclusive breastfeeding in Uganda: implications for scaling‐up. Matern Child Nutr.

[38] Cooley LVR (2012). Scaling Up - From Vision to Large‐Scale Change: A Management Framework for Practitioners.

[39] Simmons R, Shiffman J, Simmons RFP, Ghiron L (2007). Scaling up health service innovations: a framework for action. Scaling up health service delivery.

[40] Harries AD, Zachariah R, Jahn A, Schouten EJ, Kamoto K (2009). Scaling up antiretroviral therapy in Malawi-implications for managing other chronic diseases in resource-limited countries. J Acquir Immune Defic Syndr.

[41] Billings DL, Crane BB, Benson J, Solo J, Fetters T (2007). Scaling-up a public health innovation: a comparative study of post-abortion care in Bolivia and Mexico. Soc Sci Med.

[42] Khatri GR, Frieden TR (2002). Rapid DOTS expansion in India. Bull World Health Organ.

[43] Rogers EM (1995). Diffusion of innovations.

[44] Bishai D, McQuestion M, Chaudhry R, Wigton A (2006). The costs of scaling up vaccination in the world’s poorest countries. Health Aff.

[45] Wilson ML, Walker ED, Mzilahowa T, Mathanga DP, Taylor TE (2012). Malaria elimination in Malawi: research needs in highly endemic, poverty-stricken contexts. Acta Trop.

[46] Curran K, Njeuhmeli E, Mirelman A, Dickson K, Adamu T, Cherutich P (2011). Voluntary medical male circumcision: strategies for meeting the human resource needs of scale-up in southern and eastern Africa. PLoS Med.

[47] Smith BJ, Tang KC, Nutbeam D (2006). WHO health promotion glossary: new terms. Health Promot Int.

[48] Norman R, Haas M (2009). Issues in the costing of large projects in health and healthcare.

[49] Neville L, O’Hara B, Milat AJ (2009). Computer-tailored nutrition interventions targeting adults: a systematic review. Health Educ Res.

[50] Glasgow RE, Emmons KM (2007). How can we increase translation of research into practice? Types of evidence needed. Annu Rev Public Health.

[51] Rychetnik L, Bauman A, Laws R, King L, Rissel C, Nutbeam D (2012). Translating research for evidence-based public health: key concepts and future directions. J Epidemiol Community Health.

[52] Yothasamut J, Putchong C, Sirisamutr T, Teerawattananon Y, Tantivess S (2010). Scaling up cervical cancer screening in the midst of human papillomavirus vaccination advocacy in Thailand. BMC Health Serv Res.

