# Supplementary material for: Narrative review of models and success factors for scaling up public health interventions
Source: Implement Sci. 2015 Aug 12;10:113. doi: 10.1186/s13012-015-0301-6 (PMC4533941; doi:10.1186/s13012-015-0301-6)
Supplement: Additional file 1: Table S1. — Summary of characteristics of studies describing concepts, theories and models of scaling up public health action. [file 13012_2015_301_MOESM1_ESM.docx]

**Supplementary Table 1:** Summary of characteristics of studies describing concepts, theories and models of scaling up public health action

| **Reference** | **Study type** | **Concepts, theories used or proposed** | **Key concepts, elements of the model or framework** | **Context** | **Success factors & important findings** |
| --- | --- | --- | --- | --- | --- |
| Bhandari et al (2008) [[12](#_ENREF_12)] | Literature review & commentary | Model for scaling up | Model involves the following steps:   - assess situation, create a policy environment conducive to action; - define roles, relationships and responsibilities of all partners; - establish agreements; - review technical support; - define program strategy; - mobilize resources; - provide training and technical assistance; - develop and use monitoring and evaluation systems; - monitor coverage and quality; - measure impact and cost; and - provide for testing novel approaches and continuing innovation. | Global Health: breastfeeding | - Political will, advocacy, enabling policies. - Championship by health ministries at national, state and district level. - Partners with defined roles, assessment of their capacity to deliver and plans to cover gaps in capacity. - Careful selection of communication channels. - Communication strategy aided by formative research, pre-testing & validated messages and tools. - Quality trainers and training centres. - Appropriate monitoring and evaluation. - Program redesign when relevant through research & analysis. |
| Bishai et al (2006) [[13](#_ENREF_13)] | Descriptive: multi-country analysis | Costing models | - Costing modelling of average cost. - Marginal cost curves determine economies and diseconomies of scale. | Global health: immunization | - Vital to collect costs data for intervention delivery - Important to measure long-run average cost increase or decrease as output is increased. |
| Edouard et al (2012)[[14](#_ENREF_14)] | Commentary | Concept: Information and Communication Technology (ICT) | - Internet and mobile based health promotion programing. | Global Health: Youth Sexual and Reproductive Health | - Information and Communication Technology (ICT) platforms are low cost and can increase access to remote populations - Successful models should be identified through program evaluation. |
| Johns & Baltussen (2004) [[15](#_ENREF_15)] | Economic modelling: case studies | Costs and cost effectiveness | - Generalized cost effectiveness analysis. - Average cost per capita. - Marginal cost analysis. | Global health | - Inclusion of non-linear cost functions for health centres, transportation and supervision costs, as well as the presence of fixed costs of establishing a health infrastructure will advance cost effectiveness modelling. - Results show changing marginal costs as predicted by the proposed economic theory. |
| Kohl et al (2003) [[16](#_ENREF_16)] | Commentary and literature review | Definition of scaling up processes  Scaling Up Management (SUM)  Framework | SUM framework includes 3 key steps:   - Step 1: developing a scaling up plan; - Step 2: establishing the preconditions for scaling up; and - Step 3: implementing the scaling up process based on the identification of factors that can promote extension and sustainability. | Global health: reproductive health | - The more process-intensive or context-specific a model is, the more participatory the scaling up process needs to be. - The more one can simplify a model without losing the basis for its effectiveness, the more feasible it is to scale it up. - There appears to be a strong reluctance on the part of most originating organizations to simplify, repackage, or relinquish control over their models for purposes of scaling them up. - Management and leadership are major factors limiting the scaling up of successful pilot activities. The leadership and management skills needed to design and operate a successful pilot program are notably different from those needed to take a model to scale or those needed to operate on a large scale. - Early planning for scaling up can significantly affect the design and implementation of pilot activities, and can materially increase the prospects for subsequent scaling up. - Design of effective procedures for documenting and disseminating processes and results, early involvement of key decision makers, and anticipation of potential adopting organization(s) are important elements in planning. |
| Kumaranayake (2008) [[17](#_ENREF_17)] | Commentary: Cost modelling | Costing models and variation  by scale | - Relationship between average cost, marginal cost and output levels. - Marginal cost curves. - Cost-modelling. | Global health: HIV interventions | - Decision-makers encounter significant uncertainties about financing and the need to understand programing costs at different scales of delivery. - Average costs may become larger or smaller   as the volume of services expand, depending on the level of coverage and type of intervention.   - Key constraints to scaling up include infrastructure investments and cost results. - Evidence suggests that cost efficiencies associated with scale may reflect different ways of delivering services at higher volumes, including lower quality outputs. |
| Larson et al (2012) [[18](#_ENREF_18)] | Descriptive: case study | Social cognitive theory.  Social marketing frameworks | Project performance framework:   - zinc formulation (formulation, regulatory steps, technology transfer, production, distribution); - research in support of zinc scale up (pre-launch, post-launch); - product promotion (providers, caregivers mass media); - health care delivery systems (Key players and partnerships, health policy, manpower, financing); and - knowledge transfer/dissemination (website, newsletter, international conference, publications). | Bangladesh: Zinc treatment of childhood diarrhoea as an adjunct to oral rehydration  solution | - Critical to the success of a scale-up campaign is the preparation of a project performance framework that clearly identifies the major project components and the activities to be performed under each. - Scale-up campaigns ideally should be repeatedly monitored for intended and unintended consequences, including rates of zinc coverage and disparities in coverage. |
| Maher et al (2007) [[19](#_ENREF_19)] | Commentary | Mathematical modelling of scaling up | Estimates of the potential impact of  scaling up interventions on prevalence, incidence and death rates by estimating levels of tuberculosis case detection and treatment outcomes from studies. | Global health: tuberculosis control | - A consensus plan based on sound epidemiological analysis with robust budget justifications presents a convincing argument for mobilizing resources needed for action. - The plan provides a means of ensuring accountability, since progress is measured against actual milestones. |
| Mangham & Hanson (2010) [[20](#_ENREF_20)] | Commentary and literature review | Scaling up concepts  Definition of scaling up processes | Key issues in scaling up include:   - costs of scaling up coverage; - constraints to scaling up; - equity and quality concerns; and - key service delivery issues when scaling up. | Global health | - ‘Scaling up’ is used primarily to describe the ambition or process of expanding the coverage of health interventions, but can also refer to increasing the financial, human and capital resources required to expand coverage. - It is important to obtain a better understanding of how to deliver priority health interventions at scale. |
| Milat et al (2013)[[21](#_ENREF_21)] | Literature review and Delphi study | Scaling up considerations  Defining scalability | Scalability considerations include:   - Effectiveness; - Reach and adoption; - human, technical and organizational - resources; - costs; - intervention delivery; - contextual factors, and - appropriate evaluation approaches. | Scale of public health interventions in high income countries | - If scalability considerations are addressed in funding, design and reporting of intervention research will advance the quality and usability of research for policy-makers.. |
| Milat et al (2014) [[22](#_ENREF_22)] | Framework | Framework for scaling up population health action | Four step process for scaling up interventions:   - Step 1.Scalability assessment - assess the suitability of the intervention or interventions for scaling up. The outcome of this assessment will determine whether the remaining steps in the guide should be followed. - Step 2. Develop a scaling up plan - create a vision of what scaling up will look like and a compelling case for action. - Step 3. Prepare for scaling up - securing resources and building a foundation of legitimacy and support for the scaling up plan. - Step 4. Scale up - main tasks that should be addressed during scaling up. | Scale of public health interventions in high income countries | - Scaling up is a learning process, and changing the scaling up plan as learning proceeds is constructive and necessary. - Despite the unpredictability of scaling up processes, a good plan can guide scaling up processes in the right direction and make success more likely. - Effective scale up requires systematic use of evidence and it is essential that data from monitoring is linked to decision-making throughout the scaling up process. - Plans for scaling up need to consider a broad range of contextual factors and balance what is desirable with what is feasible. |
| Morel et al (2005) [[23](#_ENREF_23)] | Economic modelling: case studies | Generalized cost effectiveness analysis | - Costs, effectiveness, and cost effectiveness of 5 malaria interventions at varying scales of implementation. | Global health: strategies to combat malaria | - Achieving malaria specific millennium development goals requires a massive scaling up of interventions in sub-Saharan Africa. - Economic modelling shows advantages of shifting resources towards artemisinin based combination treatment, as well as of using preventive and curative interventions in combination. - A much larger infusion of resources than those currently available, and more attention to health system strengthening is needed to make headway in the fight to roll back malaria. |
| Norton & Mittman (2010) [[24](#_ENREF_24)] | Descriptive: interviews | Scaling up concepts | Key barriers and facilitators to  scale-up of health promotion and prevention programs. | High income countries: Health Promotion & Disease  Prevention | - Programs that are flexible and easily adapted to different target populations, implementation settings, and contexts are more likely to be widely adopted than those that are very rigid and require strict adherence to program content and components. - Programs should be flexible and amenable to rapid changes in response to: (a) participants’ feedback and (b) outcome monitoring and evaluation data. - Program materials should be available online and easily accessible. - Programs should be branded and marketed so that they are visible to potential participants and end-users. - Commitment by multiple organizations and stakeholder groups provides support and legitimacy for implementing a program. - In addition to highlighting program efficacy, it is important to a) identify and b) explicitly state the benefits of the program to potential implementers and target organizations. - Importance of partnering with an agency that has local implementation support and access to the community, in addition to having regional- or national-level infrastructure. - Funding agencies should consider adapting the grant review process to dissemination and implementation research, and expediting the grant application process for follow-up studies needed to facilitate scale-up. |
| Pearson & Ljungqvist (2011) [[25](#_ENREF_25)] | Descriptive: case study | Renewed Efforts Against Child Hunger (REACH) implementation framework | - REACH assists governments and development partners analyze the current coverage of critical interventions, prioritize nutrition actions, develop a national plan of action, and establish the required multi-sectoral governance and management mechanisms for scaling up.   REACH program is underpinned by a series of implementation and evaluation indicators including:   - key program inputs and outputs; - steps/activities for intervention delivery; - monitoring of coverage of the target population (Reach); and - indicators to measure depth and/or quality of intervention. | Global health: priority nutrition interventions | REACH framework facilitates multi-stakeholder coordination and accountability at the country level by:   - using monitoring and evaluation to help establish consensus on priorities, goals, and action among relevant stakeholders; - developing robust inputs and evidence base for decision-making, advocacy, and resource mobilization; and - diagnosing critical success factors and areas for improvement to facilitate responsive action and a continuous improvement. |
| Pérez-Escamilla et al (2012) [[26](#_ENREF_26)] | Commentary: literature review | Scaling up concepts Breastfeeding gear model | Breastfeeding gear model has 8 interrelated elements:   - advocacy; - political will; - legislation & policies; - funding and resources; - training &delivery; - promotion; - research & evaluation; and - co-ordination and goals monitoring.   Framework suggests successful multiple feedback loops and several potential paths are required to achieve intended innovation. | Global health: breast feeding | - Scale up requires a high degree of inter-sectoral coordination, usually at the national and local levels, based on a flexible decentralized structure, and sustainability requires the availability of low-cost and rapid-response monitoring and evaluation systems. - Once key user groups successfully adopt new practices in a community or region, it then becomes more likely that the use of the innovation will spread successfully to other communities through common social networks. - Failure to scale up may be attributable to insufficient assessment of user groups in context, lack of fit of the innovation with user receptivity, inability to address resistance from stakeholders, and inadequate engagement with user groups. |
| Rani et a (2012) [[27](#_ENREF_27)] | Descriptive: case study analysis | Conceptual framework for governance of responses to non-communicable diseases (NCD) | Conceptual framework to analyse governance of scaled up responses to NCDs include:   - global and regional governance processes; - national oversight, planning, management and monitoring structures - governance of NCDs in health systems; - multi-sectoral co-ordination and partnership; and - national policies, strategies and action plans. | Global health: five low and middle income countries | - Multi-sectoral coordination must be resourced to be effective. - Sector-wide national planning bodies should ensure that NCD-specific plans are:   - within the resources offered by health sector;   - include information on the scope financial commitments; and   - have realistic quantifiable targets.   - Resources to strengthen institutional capacity to respond to NCDs should not be solely targeted to NCD-specific units, but more importantly to sector- wide organizational units to build their capacity in effectively incorporating NCD-specific requirements in human resources development, health financing, medical supply and logistic and information systems. |
| Subramanian et al (2011) [[28](#_ENREF_28)] | Systematic review | Examination of conceptual models for scaling up health services in developing countries | - Models for scaling up health services place importance on enhancing organizational, functional, and political capabilities through experimentation and adaptation of strategies in addition to increasing the coverage and range of health services. - These scaling up approaches focus on fostering sustainable institutions and the constructive engagement between end users and the provider and financing organizations. | Global health: health services | - Current approaches to scaling up health services to reach the Millennium development goals are overly simplistic and not working adequately. - There is a need for feedback mechanisms to provide transparency to all players, and not just managers, on the successes and challenges in scaling up a particular strategy. - Rather than relying on blueprint planning and costing that is prevalent among global health initiatives, a “learning by doing” approach that engages key stakeholders, uses data to address constraints, and incorporates results from pilot projects, is a more promising approach to finding pathways to scaling up. |
| Underhill et al (2010) [[29](#_ENREF_29)] | Commentary: Literature review | Implementation processes that may influence the effectiveness of pre-exposure prophylaxis for public use | Proposed implementation processes include:   - scientific groundwork; - regulatory and policy groundwork; - stakeholder and infrastructure groundwork; - actual delivery; and - long-term process of monitoring and adjustment. | Global health: pre-exposure prophylaxis | - The proposed conceptual framework offers a taxonomy of implementation concerns and future work should re-examine and expand on these categories and subcategories. |
| Victora et al (2012) [[30](#_ENREF_30)] | Commentary: literature review | Scaling up barriers and enablers  Framework for scaling up | Key elements of successful scaling up of maternal nutrition programs at the country level include:   - visibility of the nutritional problem; - political will; - leadership and coordination; - partnerships including public private, and civic sectors; - technical capacity for design and implementation; - supporting policies; - program design; - financing and costing; - procurement of commodities; - strength of implementation; - consumer demand creation; - monitoring and evaluation; - sustainability; and - equity. | Global health: maternal nutrition | - Programs differ in complexity, and key elements for success vary with the type of program and the context in which they operate. - Special attention must be given to equity, as even with improved overall coverage and impact inequalities may even be increased. - Much greater investments are needed in independent monitoring and evaluation. |
| World Health Organiza-tion (WHO) (2010) [[7](#_ENREF_7)] | Framework | Proposed framework developing a scaling up strategy | ExpandNET Framework involves 9 steps:   - planning actions to increase the scalability of the innovation; - increasing the capacity of the user organization to implement; - assessing the environment and planning actions to increase the potential for success; - increasing the capacity of the resource team to support scaling up; - making strategic choices to support vertical scaling up (institutionalization); - making strategic choices to support horizontal scaling up (expansion/ replication); - determining the role of diversification; - planning actions to address spontaneous scaling up; and - finalizing the scaling-up strategy and identifying next steps. | Global Health | - Ensure the relevance of the proposed innovation. - Reach consensus on expectations for scale-up. - Tailor the innovation to the socio-cultural and institutional settings. - Keep the innovation as simple as possible. - Test the innovation in the variety of socio-cultural and institutional settings where it will be scaled-up. - Test the innovation under the routine operating conditions and existing resource constraints of the health system. |
| Willey et al (2012) [[31](#_ENREF_31)] | Commentary: Literature review | Scaling up barriers and enablers | Describes strategies for delivering ITNs at scale, with a particular focus on:   - study quality; - studies with high ITN ownership or use; - equity of ITN ownership and use; - costs; and - evaluation approaches. | Global health: insecticide-treated nets (ITN) for malaria | - Barriers involving cost were common at the user level, whereas barriers involving stock-outs and poor logistics for ITN procurement and transport were common at the implementer level. - Effective training and supervision of staff was highlighted as facilitators at the implementer level. - The evaluation of large-scale health programs is a priority using a range of real world designs. |
| Winfrey et al (2011) [[32](#_ENREF_32)] | Mortality Modelling | Modelling tools to estimate the  mortality and stillbirth impact of scaling up proven maternal and child health interventions. | Lives Saved Tool, (LiST) calculates the effects of health interventions on neonatal, child and maternal mortality. Key outputs for each type of mortality are:   - the mortality rates (neonatal, infant, under five and   maternal mortality rates);   - number of deaths; - number of still births; - number of deaths by cause; - number of deaths averted; - number of deaths averted by cause; and - number of deaths averted by intervention.   LiST also provides outputs related to nutrition. All indicators are available for each year of the projection. | Global health: maternal and child health | - LiST provides planners and policy makers with a tool to examine the potential impact of alternative strategies to reduce mortality. - It is intended to support the strategic analysis of alternatives by displaying the cause of death structure and producing output showing not only the total impact on mortality but also the contribution of each intervention to the total impact. |
| Yamey 2011 [[9](#_ENREF_9)] | Commentary: literature review & interviews | Proposed framework for scaling up Diffusion of Innovation | Describes 6 components:   - attributes of the specific tool or service being scaled up; - attributes of the implementers; - chosen delivery strategy; - attributes of the ‘‘adopting’’ community; - socio-political context & - research context. | Global health | - A key lesson learned from successful scale-up efforts is that large-scale implementation is more likely if the intervention being scaled up is simple and technically sound and there is widespread consensus about its value. - Chances of success are likely to be increased by strong leadership and governance and the active engagement of a broad range of implementers, including non-state actors. - There is no single or straightforward delivery strategy that offers a formula for success. - The field of implementation science would be advanced if scale-up efforts were always accompanied by research. |
| Zhang et al (2012)[[33](#_ENREF_33)] | Epidemiologic analysis and forecasting | Modelling the epidemiological impact of scaling up HIV  testing and antiretroviral treatment (ARVT) | - Mathematical model to simulate HIV transmission in the Chinese population over the past decade. - The model is calibrated using established biological and clinical data from the published literature, and a wide range of Chinese epidemiological and behavioral data sources. | Global health: HIV testing and ARVT | - Model provides reasonable estimations and projections of the HIV/AIDS epidemic in the future based on actual epidemiological and clinical effectiveness data. - A 10-fold increase in the treatment rate could decrease the number of HIV-related deaths by 58% and the number of new infections by one-quarter by 2015. |
